# Unravelling the Influence of Composition and Heat Treatment on Key Characteristics of Dairy Protein Powders Using a Multifactorial Approach

**DOI:** 10.3390/foods12173192

**Published:** 2023-08-24

**Authors:** Jeehyun Lee, François Martin, Emeline Goussé, Anne Dolivet, Françoise Boissel, Arnaud Paul, Jennifer Burgain, Gaëlle Tanguy, Romain Jeantet, Cécile Le Floch-Fouéré

**Affiliations:** 1 INRAE, Institut Agro, STLO, 35042 Rennes, France; jeehyun.lee@institut-agro.fr (J.L.); francois.martin@inrae.fr (F.M.); emeline.gousse@inrae.fr (E.G.); francoise.boissel@institut-agro.fr (F.B.); gaelle.tanguy@inrae.fr (G.T.); romain.jeantet@institut-agro.fr (R.J.); 2 Centre National Interprofessionnel de l’Economie Laitière (CNIEL), 75314 Paris, France; arnaud.paul@univ-lorraine.fr; 3 Laboratoire LIBio, Université de Lorraine, 54000 Nancy, France; jennifer.burgain@univ-lorraine.fr

**Keywords:** dairy powder, protein ratio, casein, whey protein, denaturation, ageing, PCA

## Abstract

The purpose of this study was to improve understanding of the structural and functional property changes that milk-protein concentrates undergo during production, particularly how the manufacturing route (heat treatment position and intensity), standardization (in osmosed water or ultrafiltrate permeate) and formulation (casein:whey protein (Cas:WP) ratio) influence the physico-chemical characteristics—hygroscopicity, particle size, sphericity, density and evolution of browning during storage. To obtain a comprehensive understanding of the parameters responsible for the distinctive characteristics of different powders, a multifactorial approach was adopted. Hygroscopicity depended mainly on the standardizing solution and to a lesser extent the Cas:WP ratio. The particle size of the heat-treated casein-dominant powders was up to 5 μm higher than for those that had had no heat treatment regardless of the standardizing solution, which also had no influence on the sphericity of the powder particles. The density of the powders increased up to 800 kg·m^−3^ with a reduced proportion of casein, and lactose and whey proteins participated in browning reactions during storage at 13 °C. In increasing order, the modality of heat treatment, the standardizing solution and the Cas:WP protein ratio influenced the key characteristics. This work is relevant for industrial applications to increase control over the functionalities of powdered products.

## 1. Introduction

Many food products are produced in dried form because the elimination of water facilitates and reduces storage and transport and guarantees food security, and dairy products are no exception. especially protein powders. These are of particular interest because they can be used as either base ingredients or complete nutritional products.

Spraying is the common drying method for producing stable dairy protein powders, but there are numerous issues in their handling and storage: powder blockage in spray dryers, powder silos and hoppers; and caking after bagging. These can be linked to particle surface composition and moisture levels, which affect key functional properties such as stickiness, wettability, bulk density and flowability [1,2]. The physico-chemical properties of spray-dried dairy powders depend first on feed composition. This influence has been studied on milk powders of various fat levels, skimmed milk powders with either added or hydrolyzed lactose, and milk-protein concentrates [1,3,4,5,6]. More recently, numerous studies have been carried out on the impact of the casein:whey protein (Cas:WP) ratio on particle shape using the single-drop or monodisperse drying technique [7,8,9].

Despite studies focused on physico-chemical and physical parameters, understanding the extent to which each factor and processing parameter influences powder characteristics remains a challenge and is of paramount importance during production. Because it can be important for engineering the quality of the final product, this paper adopts a multifactorial approach to study and discuss the influence of composition (Cas:WP ratio, lactose and mineral content) and heat treatment prior to drying (position in the technological scheme and intensity) on the key characteristics of powders.

## 2. Materials and Methods

### 2.1. Preparation of Standardized Protein Solutions

The protein solutions were prepared according to the procedure described in Martin et al. (2022) [10] using WP concentrate (27% dry matter *w*/*w*, 95% protein on dry matter) and native phosphocaseinate concentrate (13% dry matter *w*/*w*, 87% protein on dry matter), provided by the French dairy industry. Both were standardized to 10% *w*/*w* of protein using osmosed water (*water-based*) or ultrafiltrate permeate (*permeate-based*) containing 60 g·kg^−1^ of dry matter, with mainly lactose (92% of dry matter *w*/*w*) and minerals (7% of dry matter *w*/*w*). They were used directly or mixed to four different Cas:WP ratios (91:9, 81:19, 43:57 and 7:93).

### 2.2. Fabrication of Protein Powders

**Figure 1:**
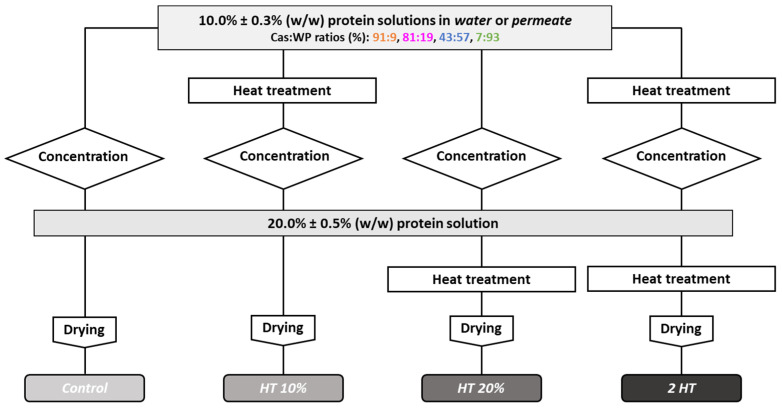
The four different process schemes for dairy protein powder production: *Control:* ■, *HT* 10%: ■, *HT* 20%: ■, 2 *HT*: ■. *Water* or *ultrafiltrate permeate* was used to standardize protein solutions at 10% *w*/*w* with cas:WP ratios (%) of 91:9, 81:19, 43:57, 7:93.

The *water-* and *permeate-based* protein solutions with different Cas:WP ratios were transformed into powders by applying heat treatment, concentrated by vacuum evaporation and spray dried (Figure 1). The *control* powder was prepared from unheated 10% *w*/*w* protein solution. Three different combinations of heat treatment were tested: 10% *w*/*w* protein solution before vacuum evaporation (*HT* 10%); 20% *w*/*w* protein solution after vacuum concentration (*HT* 20%); and before and after vacuum evaporation (2 *HT*).

Heat treatment of standardized protein solutions was carried out in a tubular exchanger (Tetra Pak, Lund, Sweden) for 30 s at 74 °C for concentrates having Cas:WP ratios of 91:9 and 81:19. The temperature was set at 70 °C for those with ratios of 43:57 and 7:93 to stand below the gelling point of the WP. The solutions were then cooled to 20 °C using a second tubular heat exchanger. The heat exchangers were composed of 11 tubes (diameter = 16 mm; length = 3 m; flow rate = 120 L·h^−1^). The detailed procedure for vacuum evaporation and spray drying is available in Martin et al. (2022) [10].

### 2.3. Sampling and Storage of Powders

Spray-dried powders were put into aluminum tins and stored at 13 °C/40% relative humidity (RH) over 12 months. The RH of the room was controlled using a dehumidifier (Blyss, Kingfisher, London, UK). During storage, the powders were sampled every two months and their water activity and browning were analyzed.

### 2.4. Determination of Chemical Composition

Before analysis, powders were rehydrated in osmosed water at 50 g·kg^−1^ protein content at room temperature for 48 h, using a stirring device (IKA Eurostar 20, Staufen im Breisgau, Germany) operating at 600 rpm. Total protein, lactose and ash content; and percentage of WP denaturation were determined following the methods described in Martin et al. (2022) [10].

### 2.5. Characterization of Physico-Chemical Properties

#### 2.5.1. Water Activity

Water activity (a_w_) was measured by a Hygrolab C1 water activity meter (Rotronic, France) at 25 °C for both fresh and stored powders.

#### 2.5.2. Dry Matter Content

One gram of powder was mixed with sand in an aluminum capsule, covered by a lid. The latter was put in an oven for 5 h at 102 °C. The dry matter (DM) content was determined by the difference in weight before and after drying.

#### 2.5.3. Water Sorption Isotherm

Water sorption isotherms were determined using an automatic sorption device (SPS, ProUmid, Ulm, Germany). Changes in the weight of powder induced by a progressive increase in RH from 0 to 95% were recorded. For each RH increment, equilibrium was reached when a change in weight was less than 0.01% between two weighing cycles (i.e., 10 min) [6]. Accurate air RH was obtained by mixing nitrogen gas and water vapor, and the measurements were conducted at 25 °C.

The Guggenheim Anderson de Boer (GAB) equation was used to model water sorption isotherms for unheated (*Control*) powders to determine water content (Equation (1)).

(1) $$m=\frac{ckRH}{\left(1-kRH)(1+kRH\left(c-1\right)\right)}\times m_{m}$$

where m as the moisture content (g·100 g^−1^ DM); RH is relative humidity; m_m_ is the monolayer value (g·100 g^−1^ DM); and c and k are constants. These latter were calculated from the parameters α, β and γ (Equations (2)–(4)) identified from the quadratic transformation of the GAB equation (Equation (5)) [11].

(2) $$m_{m}=\frac{1}{\sqrt{\beta^{2}-4\alpha \gamma }}$$

(3) k=β−(1mm)−2γ

(4) $$c=\frac{1}{m_{m}k\gamma }$$

(5) HRm=αHR2+βHR+γ

### 2.6. Characterization of Particle Size and Sphericity

#### 2.6.1. Particle Size Distribution

Particle size distribution was determined by laser light scattering (Mastersizer 2000, Malvern instruments, Malvern, UK) equipped with a 5 mW He–Ne laser operating at a wavelength of 633 nm. Air dispersion was performed by the Scirocco 2000 module (Malvern instruments, Malvern, UK). Air pressure was set at 0.5 bar and the feeding rate was between 50 and 70% as a function of powder cohesiveness [12].

#### 2.6.2. Particle Sphericity

Particle sphericity was determined using a dynamic image analysis apparatus (QICPIC, Sympactec Inc., Clausthal-Zellerfeld, Germany) equipped with a pulse laser excited at 532 nm having a pulse duration of less than a nanosecond. As the particles passed in front of the laser, they were optically “frozen” while a high-resolution camera projected their shape onto the lens (M6 lens (8.4–1877 μm)) at 500 frames per second. The air pressure to disperse the particles was 1 bar, and the feeding rate was 50%. To avoid any artefacts due to the presence of small particles or dust, particles with a diameter higher than 15 and lower than 180 μm were taken into account. More than one million particles were analyzed at each run, ensuring reliable statistical analysis. The sphericity shape factor was chosen to describe particle morphology [7]. The sphericity corresponded to the ratio of the perimeter of the equivalent circle to the real perimeter of the particles. A value of 1 depicted a perfect sphere, whereas values close to 0 represented particles with very irregular shapes.

### 2.7. Determination of Bulk Density and Compressibility

The bulk density (ρ_b_) and the tapped density (ρ_t_) were obtained using Powder Tester PT-X2 (Hosokawa Micron, Summit, NJ, USA). ρ_b_ was defined as the weight of a defined volume of powder without any compaction, and ρ_t_ was determined after a compaction step that removed air from between the particles. Compressibility was calculated using Equation (6) [13].

(6) $$Compressibity(\%)=\frac{\rho_{t}+\rho_{b}}{\rho_{t}}\times 100$$

### 2.8. Determination of Browning Index

A CR-400 chromameter (Konica Minolta, Tokyo, Japan) was used to measure the color of powder according to L*a*b* color space coordinates. L* corresponds to brightness and ranges from 0 for black to 100 for white; a* varies from −60 for green to +60 for red, and b* from −60 for blue and +60 for yellow [13]. The browning index (BI) was determined according to Equations (7) and (8) [14,15] for powders during storage between 0 and 12 months at 13 °C.

(7) $$BI=\frac{100\times (x-0.31)}{0.17}$$

(8) $$x=\frac{a^{*}+1.75\times L^{*}}{5.645\times L^{*}+a^{*}-3.012\times b^{*}}$$

Color measurements were conducted both on fresh and stored powders.

### 2.9. Statistical Analysis

All measurements were conducted in least triplicate, and the results were presented as mean ± standard deviation. Analysis of variance (one-way ANOVA, LSD test) was carried out using R (The R Foundation 2014) with a significance level of *p* < 0.05. Principal component analysis (PCA) was performed using the FactoMineR and Factoshiny packages [16]. The dataset was composed of 31 variables and 84 individuals (triplicate of the 28 studied samples). Only the *permeate-based* powders with a Cas:WP ratio of 91:9 were not considered for analysis. Variables “Hyg_10; Hyg_20 …” corresponded to the hygroscopicity values of the powders (in g·100 g^−1^ DM) extracted from the sorption isotherms at different RHs. Variables “Sph_0.0–0.5; Sph_0.5–0.6 …” represented the percentage of particles according to their sphericity ranging from less than 0.5 to more than 0.9. BI refers to the browning index of the powders.

## 3. Results and Discussion

### 3.1. Principal Component Analysis

**Figure 2:**
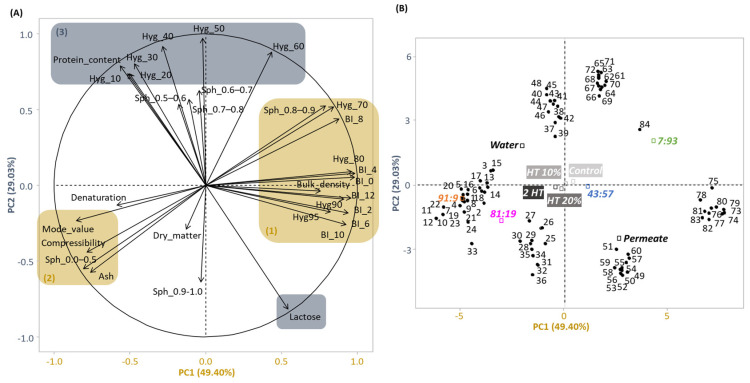
(**A**) Projection of variables according to the first two principal components PC1 and PC2 of principal component analysis: four distinctive groups of variables (1), (2), (3) and (4). Sph, Hyg and BI correspond to sphericity, hygroscopicity and Browning index respectively. The correlation between the variables is shown by the angle between their projections. (**B**) Graph of individuals, projected following their similarities according to different process schemes (*Control:* ■, *HT* 10%: ■, *HT* 20%: ■, 2 *HT*: ■) from solutions with cas:WP protein ratios (%) of 91:9, 81:19, 43:57, 7:93 (standardized with *water* or *permeate*).

PCA was performed to obtain a comprehensive understanding of parameters responsible of the distinctive features/characteristics of different powders. Figure 2A shows the variables associated with PC1 and PC2, which, respectively, accounted for 49.40 and 29.03% of the total variance. Other PCs each accounted for less than 8%. Therefore, only PC1 and PC2 were considered. Figure 2B presents the corresponding biplot of individuals where proximity reflected similarity. The PCA suggested four distinctive groups of variables (Figure 2A):

(1) variables corresponding to browning (BI), hygroscopicity at high RH (Hyg_70, Hyg_80, Hyg_90, Hyg_95), bulk density and sphericity comprised between 0.8 and 0.9 (Sph_0.8–0.9);
(2) variables related to ash content, particle size (Mode_value), lower values of sphericity (Sph_0.0–0.5) and compressibility;
(3) variables corresponding to protein content and hygroscopicity at lower RH (Hyg_10, Hyg_20, Hyg_30, Hyg_40, Hyg_50, Hyg_60);
(4) a single variable (Lactose).

Groups (1) and (2), respectively, correlated positively and negatively with PC1. (3) correlated positively with PC2, whereas (4) correlated rather negatively with PC2 (Figure 2A). Neither PC1 nor PC2 discriminated the four processing schemes. PC1 discriminated four Cas:WP ratios and PC2 discriminated two standardizing solutions (Figure 2B). Compared to the WP-dominant powders, Cas-dominant powders had higher compressibility (Figure S1, Supplementary Materials), particle size, ash content but lower bulk density, sphericity, browning index, water uptake capacity at RH > 70%. *Water-based* powders contained higher protein content and hygroscopicity at RH < 50% and a lower level of lactose than *permeate-based* powders.

Among the three factors (Cas:WP ratio, standardizing solution and process scheme), the Cas:WP ratio was the most influential on all of measured powder characteristics except for hygroscopicity. The standardizing solution was found to be a non-negligible factor on hygroscopicity. The process scheme as implemented in this study had little influence on the physical properties of the powders. In the following sections, only variables judged relevant are presented and discussed.

### 3.2. Chemical and Physico-Chemical Composition of the Powders

Table 1 summarizes the DM content, a_w_, protein, lactose and ash content of the powders. The DM content was of the same order of magnitude for all: between 94.03 ± 0.10 and 96.09 ± 0.12 g·100 g^−1^ of powder; and a_w_ was low, ranging from 0.10 ± 0.00 to 0.16 ± 0.00. There was no significant influence of the Cas:WP ratio, standardizing solution or process scheme on DM content or a_w_ although protein content was higher in *water-based* than *permeate-based* powders due to the presence of minerals and lactose. The lactose content was higher in *permeate-based* powders as expected. It also increased along with WP content except for 43:57 powders. The ash content was higher for casein micelle (CM)-dominant and *permeate-based* powders.

**Figure 3:**
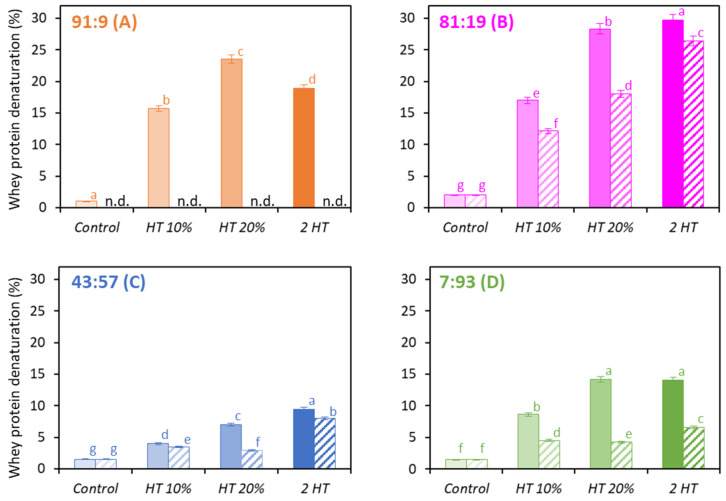
Level of whey protein denaturation (%) for the *water-based* (full bars) and *permeate-based* (hatched bars) powders with Cas:WP ratios (%) of 91:9 (**A**), 81:19 (**B**), 43:57 (**C**), 7:93 (**D**), heat-treated differently (*n* = 3–6). n.d.: not determined. Same letters within a Cas:WP ratio indicate no significant difference (*p* < 0.05).

The level of WP denaturation was assessed for all powders (Figure 3). Concentration by vacuum evaporation and spray drying induced very low levels of WP denaturation. Unheated powders (*Control*) had the lowest levels (<2%) [17,18,19,20].

The higher the concentration of protein when heat treated, the higher the WP denaturation: *HT* 20% powders contained a higher amount of denatured WP compared to *HT* 10% powders. This was also observed by Wolz and Kulozik (2015) [21] and Ho et al. (2019) [22], who suggested that the higher concentration of protein was expected to increase the collision probability between molecules; hence, the denaturation. The more intense the heat treatment, the higher the level of denaturation except for *water-based* powder with the Cas:WP ratio of 91:9 (Figure 3A). Such an increased heat-treatment effect on denaturation was not cumulative: the level of WP denaturation in 2 *HT* powders was not equal to the sum of the levels of *HT* 10% and *HT* 20% powders. The first heat treatment at 10% protein content induced protein denaturation and led to the formation of protein aggregates. Thus, less native WPs were available during the second heat treatment at 20% protein content because the reaction was deprived of a part of its reactive material (i.e., native whey proteins), resulting in fewer collisions and less aggregation.

In *water-based* 91:9, 81:19 and 7:93 powders (Figure 3A,B,D), the *HT* 20% scheme induced a similar or higher amount of denatured WP than did the 2 *HT* scheme. All *permeate-based* powders had a lower level of denaturation compared to *water-based* powders, which may be explained by the higher quantity of lactose, which is known to have a protective effect on the denaturation/aggregation of WP [23,24]. The difference in the level of denaturation between *water-* and *permeate-based* powders was higher for *HT* 20% than for *HT* 10%. There could be a threshold effect related to total protein concentration: lactose–protein interactions may not be sufficient to exert a protective effect at 10% *w*/*w*, whereas it may be effective at 20% *w*/*w* since the molecules in the system are more packed, providing steric hindrance. Cas-dominant powders showed higher levels of denaturation than WP-dominant powders, which can be explained by the difference in heat treatment temperature (74 vs. 70 °C). A rise in the proportion of Cas from 81% (Figure 3B) to 91% (Figure 3A) and from 7% (Figure 3D) to 43% (Figure 3C) resulted in a decrease in WP denaturation, which can be explained by the chaperone character of Cas with respect to the unfolding of WP induced by heating [24,25,26].

### 3.3. Water Sorption Isotherm and Hygroscopicity

**Figure 4:**
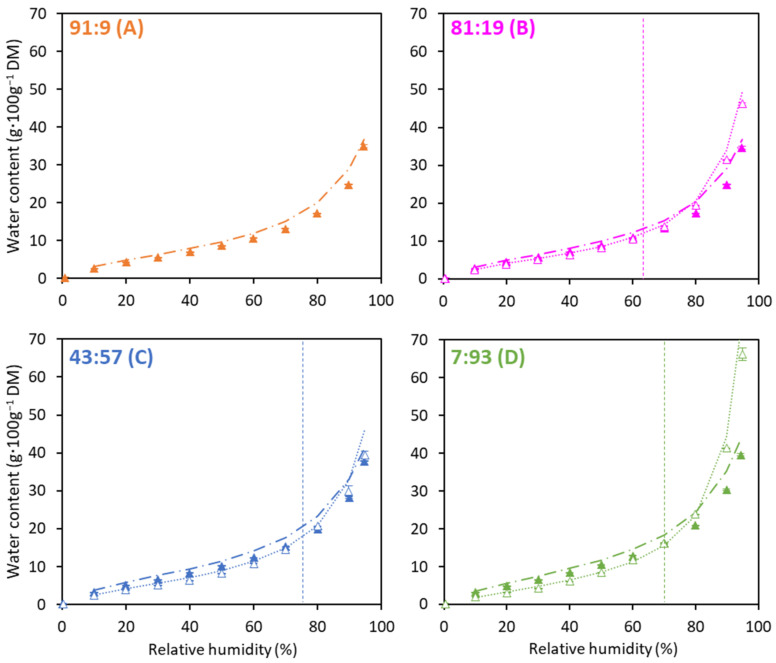
Water sorption isotherms for the *water-based* (full symbols) and *permeate-based* (empty symbols), unheated (*Control*) powders with Cas:WP ratios (%) of 91:9 (**A**), 81:19 (**B**), 43:57 (**C**), 7:93 (**D**) (*n* = 3–6). Indication of inversion of *the water-* and *permeate-based* curves (----). GAB equation fitted to *water-based* experimental data (−⋅−⋅) and *permeate-based* experimental data (⋅⋅⋅⋅).

Water sorption isotherms of the powders measured at different values of RH are presented in Figure 4. Only the curves corresponding to *Control* powders are displayed because all sorption isotherms overlapped regardless of process scheme. They all showed sigmoidal behavior, reflecting a Type II isotherm.

This result differed from that of Gianfrancesco et al. (2011) [27], who found different sorption isotherms for native β-Lg powder and denatured β-Lg powder. This difference could be explained by the much lower level of denatured β-Lg in our powder (<30%) compared to their work. The isotherm data of the powders were in good agreement with the literature, especially for the ratio 91:9 compared to micellar casein powders [28,29,30,31,32]. Gaiani et al. (2009) [32] reported a water uptake value of 23 g·100 g^−1^ DM at 90% (RH) similar to the value obtained in this work (25 g·100 g^−1^ DM) (Figure 4A). Moreover, the higher the proportion of WP, the more hygroscopic the powders. This was consistent with the findings of Ji et al. (2016) [33], who observed that WP-isolate powders were more hygroscopic than milk-protein isolate and micellar-casein powders. In our study, *water-based* powders were the more hygroscopic until 60, 75 and 70% RH for powders with Cas:WP ratios of 81:19, 43:57 and 7:93, respectively. Such an inversion of the *water-* and *permeate-based* curves suggested that until the inversion points (% RH), the proteins were the main absorber of water, which was in agreement with the findings of Berlin et al. (1968), [28] who reported that macromolecular molecules (proteins) absorbed water mainly below 50% RH, whereas lactose and minerals were the most active molecules in water uptake above 50% RH. McCarthy et al. (2013) [34] also found that below 50% RH, an increase in the protein content of dry infant milk formula induced a greater amount of water uptake, confirming that proteins dominated sorption behavior in this RH range. Same behavior was reported by Kelly et al. (2015) [6] in milk-protein concentrate powders. Over a full sorption cycle (0–95% RH), *water-based* powders absorbed less water than did *permeate-based* powders. This difference was particularly noticeable for the 7:93 powders, where there was a water uptake of 40 and 66 g·100 g^−1^ DM at 95% RH, respectively. Differences in ash content (minerals) and lactose (Table 1) between *water-* and *permeate-based* powders could explain this result.

The GAB model was in a good agreement with experimental data (Table 2) as the GAB constants were of the same order of magnitude as in the literature [27]. The monolayer capacity (m_m_) was higher when the standardizing solution was water and when WP was in higher proportion. The C values that referred to the water binding energy to the monolayer followed the same trend as m_m_, confirming the above discussion concerning hygroscopicity.

### 3.4. Particle Size and Sphericity

**Figure 5:**
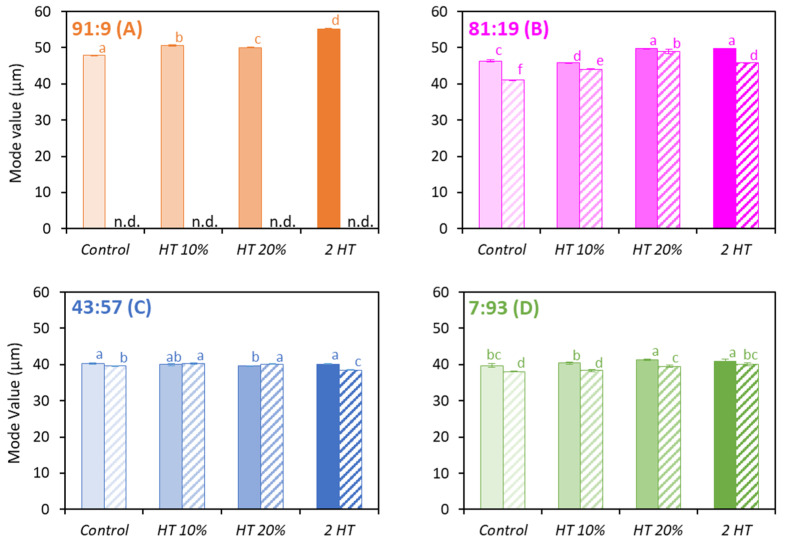
Mode value (μm) for the *water-based* (full bars) and *permeate-based* (hatched bars) powders with Cas:WP ratios (%) of 91:9 (**A**), 81:19 (**B**), 43:57 (**C**), 7:93 (**D**), heat-treated differently (*n* = 2–3). n.d.: not determined. Same letters within a Cas:WP ratio indicate no significant difference (*p* < 0.05).

Size measurements were displayed as the mode value (diameter corresponding to the maximum percentage of the sample, at the top of the peak) in Figure 5. The sizes were compatible with the ones from the literature [13,35]. The CM-dominant powders (Figure 5A,B) had particles of a larger diameter compared to the WP dominant powders (Figure 5C,D). Among the *Control* powders, those *water-based* with the Cas:WP ratio of 91:9 had the largest size, whereas the *permeate-based* with a ratio of 7:93 had the smallest particles, respectively, 48 and 38 μm. This difference can be explained by a much higher viscosity of the concentrates with the ratio 91:9 compared to the one with the ratio 7:93. Indeed, a casein is a much larger entity than a WP, which produced a greater flow resistance [36]. The *water-based* powders generally had larger particles than the *permeate-based* powders. Figure 4 also shows that the heat-treated CM-dominant powders (Figure 5A,B) had larger particles than those that did not have heat treatment. For example, the 2 *HT* powder with the Cas:WP ratio of 91:9 had a mean particle size 15% higher than for the *Control* powder (Figure 5A). It can thus be assumed that the heat treatment induced a significant increase in the volume fraction of the concentrates before spray drying [36]. Indeed, the denatured WPs had a greater water retention capacity than the native ones [37], and CMs coated with denatured WP had increased volume [38]. The viscosity of these concentrates consequently increased and led to the formation of larger droplets when spraying the product, and therefore larger particles of powder. On the other hand, less difference was observed between WP-dominant powders with or without heat treatment (Figure 5C,D), as the volume fraction of WP was much lower than that of CMs. Moreover, despite a certain level of WP denaturation (Figure 3), it can be suggested that it was not enough to increase the volume fraction or the subsequent viscosity of the feed product significantly, which could have induced a difference in particle size after spray drying.

**Figure 6:**
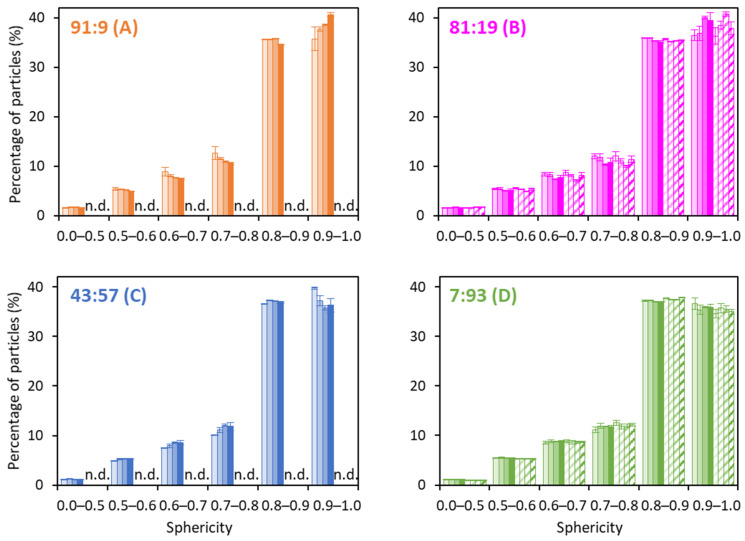
Sphericity for the *water-based* (full bars) and *permeate-based* (hatched bars) powders with Cas:WP ratios (%) of 91:9 (**A**), 81:19 (**B**), 43:57 (**C**), 7:93 (**D**), heat-treated differently (*Control*: ■, *HT* 10%: ■, *HT* 20%: ■, 2 *HT*: ■) (*n* = 3–6). n.d.: not determined.

Percentages of particles as a function of their sphericity ranged from less than 0.5 and more than 0.9 as shown in Figure 6. There was no significant difference in the sphericity between *water-* and *permeate-based* powders (Figure 6B,D). The influence of protein composition itself on the sphericity of powders was not significant. Sadek et al. (2015) [39] analyzed the morphology of powders produced in a monodisperse spray drier and demonstrated that sphericity decreased at higher casein proportions. They showed that pure casein powders presented particles with many invaginations, folded in on themselves, whereas WP-dominant powders were more likely to present smoother and more spherical particles. Gaiani et al. (2007) [35] reported that industrial powders showed a wide range of sizes and shapes making it difficult to establish a relationship between protein composition and particle shape. Yu et al. (2021) and Lanotte et al. (2018) [8,18] suggested that differences in morphologies observed for different Cas:WP ratios were due to differences in the mechanical properties of the skin formed on the surface of the drop during spray drying. WPs make a particle skin with greater resistance to deformation forces in comparison to those of CMs. In CM-dominant powders, heat treatment clearly resulted in the formation of more spherical particles (Figure 6A,B), whereas the opposite trend was observed for WP-dominant powders (Figure 6C,D). This suggested that heat treatment induced an improvement in the mechanical strength of the protein film (skin) formed on the CM-dominant drop surface during drying. The protein film was then more resistant to the deformation forces that occurred on the surface of the drop during water removal, leading to the formation of smoother particles. The mechanical strength of the skin may have been enhanced due to the presence of denatured β-Lg at the surface of the CMs, which formed connections between them, leading to a more rigid protein network. Another explanation could be increased viscosity induced by heat treatment, leading to the formation of larger droplets containing large amount of material, giving them a better ability than smaller droplets to resist deformation forces. In an opposite manner, in WP-dominant powders, it is possible that heat treatment induced the formation of soluble denatured β-Lg aggregates by modifying the mechanical properties of the skin. Malafronte et al. (2019) [40] reported that the drying of a solution of soluble whey-protein aggregates led to the formation of shriveled particles.

### 3.5. Density

**Figure 7:**
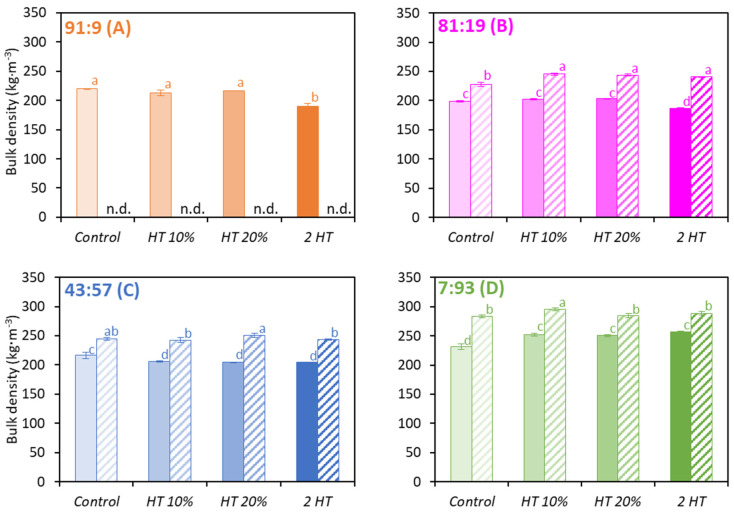
Bulk density of the *water-based* (full bars) and *permeate-based* (hatched bars) powders with Cas:WP ratios (%) of 91:9 (**A**), 81:19 (**B**), 43:57 (**C**), 7:93 (**D**), heat-treated differently (*n* = 3–6). n.d.: not determined. Same letters within a Cas:WP ratio indicate no significant difference (*p* < 0.05).

Bulk densities are presented in Figure 7. The experimentally determined values were of the same order of magnitude as the ones from the literature [13,41]. There was no significant difference among the 91:9, 81:19 and 43:57 powders. However, when WP was predominantly present (Figure 7D), bulk density was higher, especially for *permeate-based* powders. (Figure 7). These results corroborated the work of Schuck et al. (2012) [13], who reported higher bulk density for whey protein powders compared to micellar casein ones. *Permeate-based* powders had a higher bulk density than the *water-based* ones. The 2 *HT* scheme induced a decrease in bulk density for *water-based* CM-dominant powders (Figure 7A,B). It can be assumed that the increase in concentrate viscosity induced by the heat treatment stabilized the air during the process, thereby inducing a higher content of air in the powder. McSweeney et al. (2022) [12] showed that heat treatment of dairy protein concentrates induced an increase in the amount of occluded air in the resulting powder compared to the untreated solution. The presence of previously observed larger particles (Figure 5) may also have led to a higher content of interstitial air. Conversely, the 2 *HT* scheme induced an increase in bulk density for *water-based* 7:93 powders (Figure 7D).

### 3.6. Browning Index during Ageing of the Powders

**Figure 8:**
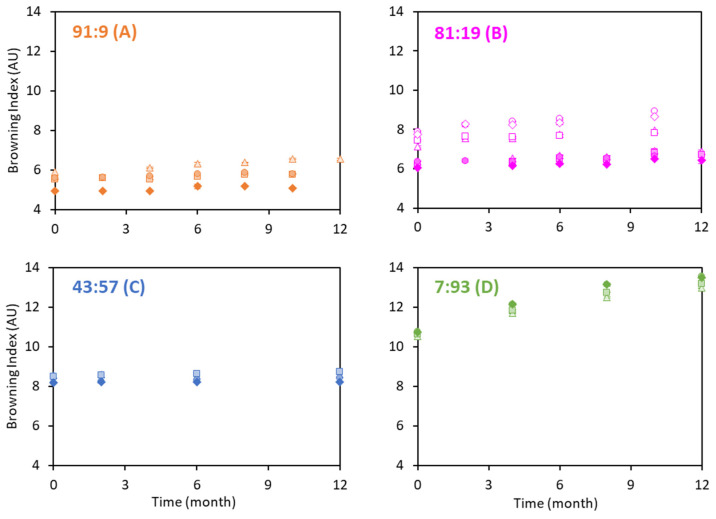
Browning index (BI) at different times during storage at 13 °C of the *water-based* (full symbols) and *permeate-based* (empty symbols) powders with Cas:WP ratios (%) of 91:9 (**A**), 81:19 (**B**), 43:57 (**C**), 7:93 (**D**), heat-treated differently (*Control*: ▲, *HT* 10%: ■, *HT* 20%: ●, 2 *HT*: ◆) (*n* = 3–6).

The BI was determined from the coordinates L*, a*, b* in the color space for the powders sampled at different times during storage at 13 °C (Figure 8). The higher the proportion of WP, the higher initial BI value, which was higher for *permeate-based* powders (Figure 8B). As expected, WPs participate in numerous browning reactions (Maillard reaction and caramelization) [42,43,44], so finding such a trend was expected. This was not explained by the lactose content, which did not follow any particular trend among the four compositions (Table 1). The difference in the Browning index for *water-* and *permeate-based* powders was not particularly significant. Indeed, Paul et al. (2022) [15] recently suggested that the index for WP powders during storage was not related to the lactose concentration but rather to the initial amount of WP lactosylation in the fresh powder. To investigate further, it will also be necessary to quantify the initial amount of lactosylation.

## 4. Conclusions

The influence of composition (Cas:WP ratio, standardizing solution) and heat treatment on key characteristics of protein powders (hygroscopicity, particle size, sphericity, density and evolution of browning during storage) was assessed. Water sorption isotherms showed that water uptake of the powders depended mainly on the standardizing solution and the Cas:WP ratio to a lesser extent. The higher the proportion of the WP, the more hygroscopic the powders. *Permeate-based* powders were less hygroscopic than the *water-based* powders until 60–75% RH. The mode values of the powders increased with the proportion of CM but varied a little between *water-* and *permeate-based* powders. There was clearly no influence of the standardizing solution on the sphericity of powder particles whereas the relationship between the Cas:WP ratio and sphericity was less obvious. The density of the powders increased with higher amounts of lactose and minerals and a reduced proportion of CM. Color measurements of powders during aging at 13 °C confirmed the participation of lactose and WP in browning reactions. PCA analysis reinforced these conclusions. In increasing order, the modality of heat treatment, the standardizing solution and the Cas:WP ratio influenced the physico-chemical properties. The results can improve predictions of the functionality of the final protein powders, and it will be interesting to investigate their rehydration properties.

## Figures and Tables

**Table 1:** Water activity, contents of dry matter, protein, lactose and ash of the powders.

Cas: WP	Standardizing Solution	Processing Scheme	Dry Matter Content (g·100 g^−1^ *)			Water Activity			Protein Content (g·100 g^−1^ *)			Ash Content (g·100 g^−1^ *)			Lactose (g·100 g^−1^ *)		
91:9	*Water*	*Control*	95.43 ^d^	±	0.14	0.14 ^h^	±	0.00	81.79 ^j^	±	0.11	8.20 ^a^	±	0.03	2.51 ^g^	±	0.01
		*HT* 10%	95.37 ^d^	±	0.03	0.13 ^p^	±	0.00	82.88 ^h^	±	0.03	7.65 ^b^	±	0.01	2.58 ^f^	±	0.04
		*HT* 20%	95.58 ^c^	±	0.14	0.14 ^i^	±	0.00	82.82 ^h^	±	0.12	7.62 ^bc^	±	0.01	2.50 ^g^	±	0.02
		2 *HT*	95.73 ^b^	±	0.08	0.14 ^l^	±	0.00	83.37 ^g^	±	0.05	7.61 ^bc^	±	0.01	2.54 ^fg^	±	0.01
81:19	*Water*	*Control*	94.99 ^gh^	±	0.11	0.13 ^m^	±	0.00	82.80 ^h^	±	0.09	7.57 ^bcd^	±	0.04	2.08 ^h^	±	0.01
		*HT* 10%	95.10 ^fg^	±	0.08	0.15 ^d^	±	0.00	82.19 ^i^	±	0.16	7.51 ^de^	±	0.05	2.08 ^h^	±	0.00
		*HT* 20%	95.03 ^g^	±	0.14	0.13 ^o^	±	0.00	83.36 ^g^	±	0.12	6.92 ^f^	±	0.03	2.09 ^h^	±	0.01
		2 *HT*	95.37 ^d^	±	0.15	0.15 ^e^	±	0.00	82.15 ^i^	±	0.12	7.55 ^cde^	±	0.01	2.08 ^h^	±	0.01
	*Permeate*	*Control*	95.32 ^de^	±	0.02	0.16 ^c^	±	0.00	73.68 ^k^	±	0.02	7.47 ^e^	±	0.02	13.98 ^e^	±	0.01
		*HT* 10%	95.21 ^ef^	±	0.03	0.14 ^k^	±	0.00	72.76 ^m^	±	0.02	7.52 ^cde^	±	0.05	13.99 ^e^	±	0.01
		*HT* 20%	95.61 ^bc^	±	0.06	0.13 ^n^	±	0.00	72.68 ^m^	±	0.05	7.50 ^de^	±	0.04	13.97 ^e^	±	0.01
		2 *HT*	95.68 ^bc^	±	0.08	0.14 ^j^	±	0.00	73.01 ^l^	±	0.06	7.48 ^de^	±	0.16	13.97 ^e^	±	0.02
43:57	*Water*	*Control*	94.87 ^h^	±	0.11	0.11 ^s^	±	0.00	84.83 ^d^	±	0.10	4.53 ^i^	±	0.44	n.d.	±	n.d.
		*HT* 10%	94.63 ^i^	±	0.07	0.10 ^t^	±	0.00	85.60 ^a^	±	0.08	4.50 ^i^	±	0.24	n.d.	±	n.d.
		*HT* 20%	94.41 ^j^	±	0.04	0.12 ^r^	±	0.00	83.28 ^g^	±	0.03	4.50 ^i^	±	0.14	n.d.	±	n.d.
		2 *HT*	94.17 ^k^	±	0.08	0.13 ^q^	±	0.00	83.50 ^f^	±	0.07	4.50 ^i^	±	0.30	n.d.	±	n.d.
	*Permeate*	*Control*	93.94 ^l^	±	0.06	n.d.		n.d.	70.39 ^o^	±	0.04	5.09 ^g^	±	0.24	17.98 ^a^	±	0.04
		*HT* 10%	94.03 ^kl^	±	0.10	n.d.		n.d.	68.25 ^t^	±	0.07	4.94 ^h^	±	0.05	17.96 ^a^	±	0.01
		*HT* 20%	94.32 ^j^	±	0.07	n.d.		n.d.	68.74 ^s^	±	0.05	4.97 ^h^	±	2.53	18.01 ^a^	±	0.01
		2 *HT*	94.42 ^j^	±	0.02	n.d.		n.d.	69.24 ^r^	±	0.01	5.03 ^gh^	±	0.45	17.97 ^a^	±	0.01
7:93	*Water*	*Control*	94.35 ^j^	±	0.06	0.16 ^a^	±	0.00	85.61 ^a^	±	0.05	1.67 ^m^	±	0.11	0.19 ^i^	±	0.01
		*HT* 10%	94.57 ^i^	±	0.06	0.16 ^b^	±	0.00	85.00 ^c^	±	0.06	1.69 ^m^	±	0.14	0.19 ^i^	±	0.01
		*HT* 20%	94.61 ^i^	±	0.02	0.15 ^f^	±	0.00	84.70 ^e^	±	0.02	1.62 ^m^	±	0.46	0.22 ^i^	±	0.02
		2 *HT*	94.70 ^i^	±	0.03	0.14 ^g^	±	0.00	85.18 ^b^	±	0.02	1.64 ^m^	±	0.08	0.18 ^i^	±	0.01
	*Permeate*	*Control*	95.62 ^bc^	±	0.01	n.d.		n.d.	69.92 ^p^	±	0.01	3.17 ^l^	±	0.52	15.25 ^b^	±	0.07
		*HT* 10%	95.57 ^c^	±	0.11	n.d.		n.d.	71.66 ^n^	±	0.08	3.28 ^jk^	±	0.51	15.15 ^c^	±	0.07
		*HT* 20%	95.67 ^bc^	±	0.08	n.d.		n.d.	70.47 ^o^	±	0.06	3.20 ^kl^	±	1.83	15.25 ^b^	±	0.07
		2 *HT*	96.09 ^a^	±	0.12	n.d.		n.d.	69.42 ^q^	±	0.09	3.36 ^j^	±	0.47	15.00 ^d^	±	0.00

* Quantity per 100 g of powder; n.d.: not determined; values are means ± analytical SD (*n* = 3–6). Same letters in a column mean no significant difference (*p* < 0.05).

**Table 2:** Identified parameters of the quadratic regression analysis and corresponding Guggenheim Anderson de Boer (GAB) monolayer value (m_m_) and isotherm constants k, c for unheated (*Control*) powders. R^2^: coefficient of determination of quadratic regression; RMSE: root mean square error as an index of quality of fit.

Cas:WP	Standardizing Solution	Quadratic Regression				GAB Model			
		α	β	γ	R^2^	m_m_	k	c	RMSE
91:9	*Water*	−0.142	0.140	0.0242	0.990	5.48	0.883	8.61	0.136
81:19	*Water*	−0.139	0.137	0.0239	0.991	5.59	0.878	8.53	0.141
	*Permeate*	−0.166	0.148	0.0290	0.995	4.93	0.947	7.38	0.0611
43:57	*Water*	−0.121	0.122	0.0194	0.993	6.43	0.873	9.16	0.147
	*Permeate*	−0.142	0.136	0.0299	0.982	5.23	0.929	6.87	0.0920
7:93	*Water*	−0.112	0.107	0.0230	0.992	7.00	0.878	7.28	0.140
	*Permeate*	−0.137	0.0918	0.0486	0.973	5.34	0.981	3.93	0.0757

## Data Availability

Data is contained within the article or Supplementary Materials.

## Supplementary Materials

The following supporting information can be downloaded at: https://www.mdpi.com/article/10.3390/foods12173192/s1, Figure S1: Compressibility (%) for the *water-based* (full bars) and *permeate-based* (hatched bars) powders with Cas:WP ratios (%) of 91:9 (A), 81:19 (B), 43:57 (C), 7:93 (D), heat-treated differently (*Control:* ■, *HT* 10%: ■, *HT* 20%: ■, 2 *HT*: ■) (*n* = 3–6). n.d.: not determined.
