# Supplementary material for: Unravelling the Influence of Composition and Heat Treatment on Key Characteristics of Dairy Protein Powders Using a Multifactorial Approach
Source: Foods. 2023 Aug 24;12(17):3192. doi: 10.3390/foods12173192 (PMC10486507; doi:10.3390/foods12173192)
Supplement: Supplementary file 1 [file foods-12-03192-s001.zip › foods-2500894-supplementary.pdf]

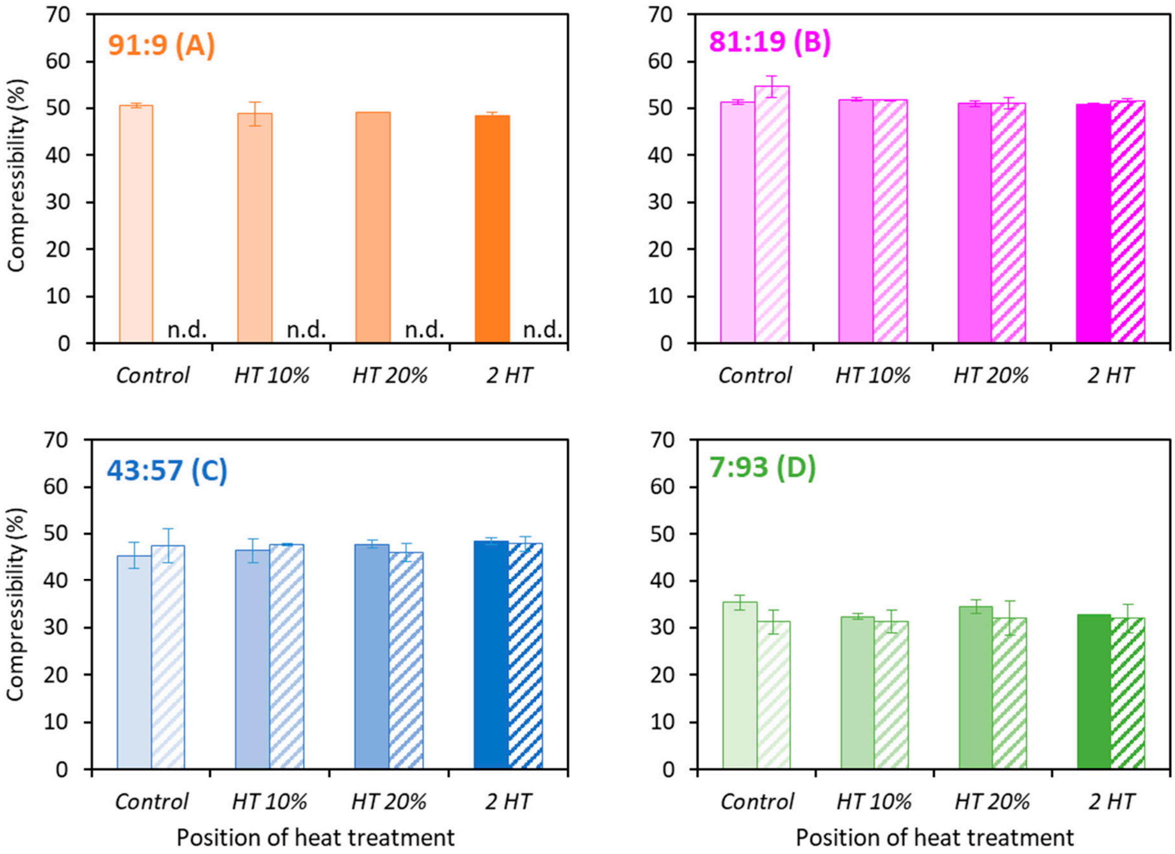

Figure S1. Compressibility (%) for the *water-based* (full bars) and *permeate-based* (hatched bars) powders with Cas:WP ratios (%) of 91:9 (A), 81:19 (B), 43:57 (C), 7:93 (D), heat-treated differently (*Control:* ■, *HT 10%*: ■, *HT 20%*: ■, *2 HT*: ■) (*n* = 3-6). n.d.: not determined.
